# Fingolimod Augments Monomethylfumarate Killing of GBM Cells

**DOI:** 10.3389/fonc.2020.00022

**Published:** 2020-01-28

**Authors:** Paul Dent, Laurence Booth, Jane L. Roberts, Andrew Poklepovic, John F. Hancock

**Affiliations:** ^1^Departments of Biochemistry and Molecular Biology, Virginia Commonwealth University, Richmond, VA, United States; ^2^Departments of Medicine, Virginia Commonwealth University, Richmond, VA, United States; ^3^Department of Integrative Biology and Pharmacology, University of Texas Health Science Center, Houston, TX, United States

**Keywords:** fingolimod, dimethyl fumarate, Gilenya, Tecfidera, RAS, glioblastoma, microglia

## Abstract

Previously we demonstrated that the multiple sclerosis drug dimethyl fumarate (DMF) and its plasma breakdown product MMF could interact with chemotherapeutic agents to kill both GBM cells and activated microglia. The trial NCT02337426 demonstrated the safety of DMF in newly diagnosed GBM patients when combined with the standard of care Stupp protocol. We hypothesized that another multiple sclerosis drug, fingolimod (FTY720) would synergize with MMF to kill GBM cells. MMF and fingolimod interacted in a greater than additive fashion to kill PDX GBM isolates. MMF and fingolimod radiosensitized glioma cells and enhanced the lethality of temozolomide. Exposure to [MMF + fingolimod] activated an ATM-dependent toxic autophagy pathway, enhanced protective endoplasmic reticulum stress signaling, and inactivated protective PI3K, STAT, and YAP function. The drug combination reduced the expression of protective c-FLIP-s, MCL-1, BCL-XL, and in parallel caused cell-surface clustering of the death receptor CD95. Knock down of CD95 or over-expression of c-FLIP-s or BCL-XL suppressed killing. Fingolimod and MMF interacted in a greater than additive fashion to rapidly enhance reactive oxygen species production and over-expression of either thioredoxin or super-oxide dismutase two significantly reduced the drug-induced phosphorylation of ATM, autophagosome formation and [MMF + fingolimod] lethality. In contrast, the production of ROS was only marginally reduced in cells lacking ATM, CD95, or Beclin1. Collectively, our data demonstrate that the primary generation of ROS by [MMF + fingolimod] plays a key role, via the induction of toxic autophagy and death receptor signaling, in the killing of GBM cells.

## Introduction

Glioblastoma multiforme (GBM) remains an incurable malignancy, with a median survival of ~14 months from initial presentation (1). There are several issues with developing new effective GBM therapeutics; e.g., the blood brain barrier often prevents the full therapeutic dose of many drugs reaching a brain-localized tumor; the brain is also an immunologically privileged environment that results both in a lack of checkpoint immunotherapy efficacy but also in an environment that contains activated brain associated macrophages, the microglia, which promote the growth, invasion, and survival of GBM cells (2–6). In the specific instance of brain tumors, whether metastatic disease or GBMs, activated microglia have a symbiotic relationship with tumor cells, each producing growth factors and cytokines that reinforces the malignant phenotype of the tumor cells (7, 8). Thus, one approach to treat tumors localized in the brain would be to break the symbiotic positive relationship between the microglia and the tumor cells.

Multiple sclerosis is a disease in which brain and spinal cord axons undergo demyelination in part due to the actions of an auto-immune disease (9, 10). Activated T cells can enter the CNS where they cause inflammation that promotes additional demyelination, that in turn attracts macrophages that enhance the inflammatory response. In the past 10 years two novel therapeutic agents have been approved for the treatment of relapsing remitting MS: Gilenya^®^ (fingolimod, FTY720) and Tecfidera^®^ (dimethyl-fumarate, DMF) (11, 12). Both drugs are administered orally (PO). Fingolimod is an analog of sphingosine-1-phosphate (S1P), with a plasma C max of ~150 nM. Fingolimod is taken up and phosphorylated before it then acts in an autocrine fashion to activate S1P receptors; receptor activation results in receptor internalization and its proteolytic destruction (13, 14). This results in reactive T cells not migrating to the site of CNS inflammation, and a reduction in disease sequelae. DMF is rapidly metabolized in the plasma of patients to monomethyl-fumarate (MMF) and has a C max in plasma of ~15 μM, with an approximate steady state tissue and plasma concentration of 5 μM. Most laboratory-based oncology studies have used DMF, not MMF, and in over 90% of all publications have used the drug above the clinically relevant range, and the range recommended by the drug makers, Biogen (15, 16). Thus, very little is known about the “real” biology of MMF. For example, high concentrations of DMF, e.g., 30–100 μM, can rapidly increase expression of the anti-oxidant enzymes NRF2 and HO-1 in tumor cells (17, 18). However, in our hands, using MMF at a clinically relevant concentration of 5 μM, no alterations in NRF2 or HO-1 expression were observed in a genetically diverse set of primary human GBM cells. At supra-physiologic concentrations DMF can suppress the inflammatory biology of microglia and astrocytes (19).

The present studies were performed to determine whether the multiple sclerosis medications fingolimod and MMF interact to kill GBM cells and to determine some of the molecular mechanisms by which killing occurs. Our data strongly argue that ATM-AMPK, CD95-caspase 8, and reactive oxygen species signaling play key roles in the killing efficacy of the drug combination.

## Materials and Methods

### Materials

MMF was purchased from Selleckchem (Houston, TX). Neratinib was kindly supplied by Puma Biotechnology Inc. (Los Angeles, CA). Fingolimod (FTY720) was purchased from Sigma-Aldrich (St. Louis MO). Trypsin-EDTA, DMEM, RPMI, penicillin-streptomycin were purchased from GIBCOBRL (GIBCOBRL Life Technologies, Grand Island, NY). Other reagents and performance of experimental procedures were as described (15, 20–24). Antibodies used: AIF (5318), BAX (5023), BAK (12105), BAD (9239), BIM (2933), BAK1 (12105), Beclin1 (3495), cathepsin B (31718), CD95 (8023), FADD (2782), eIF2α (5324), P-eIF2α S51 (3398), ULK-1 (8054), P-ULK-1 S757 (14202), P-AMPK S51 (2535), AMPKα (2532), P-ATM S1981 (13050), ATM (2873), ATG5 (12994), mTOR (2983), P-mTOR S2448 (5536), P-mTOR S2481 (2974), ATG13 (13468), MCL-1 (94296), BCL-XL (2764), P-AKT T308 (13038), P-ERK1/2 (5726), P-STAT3 Y705 (9145), P-p65 S536 (3033), p62 (23214), LAMP2 (49067) all from Cell Signaling Technology; P-ULK-1 S317 (3803a) from Abgent; P-ATG13 S318 (19127) from Novus Biologicals.

### Methods

#### Culture, Transfection and *in vitro* Exposure of Cells to Drugs

Primary human GBM isolates were grown in bulk in the flanks of NRG mice; multiple tumor isolates were used throughout the studies in this manuscript. Briefly, tumors were isolated, mechanically macerated, filtered and plated in flasks. Initially, cells were cultured at 37°C (5% (v/v CO_2_) *in vitro* using RPMI supplemented with 0.5% (v/v) fetal calf serum and 10% (v/v) Non-essential amino acids. After ~2 weeks of growth and several passages to remove contaminating mouse fibroblasts, GBM cells were grown in RPMI supplemented with 2.0% (v/v) fetal calf serum and 10% (v/v) Non-essential amino acids. Cells were frozen down in bulk and each vial grown/utilized for a maximum of four weeks of *in vitro* culture. Stem cell variants of the PDX GBM isolates were prepared as described (15, 25–27). Freshly isolated GBM cells and activated microglia directly from the operating room were separated and grown in RPMI supplemented with 2.0% (v/v) fetal calf serum and 10% (v/v) Non-essential amino acids for 6 h, followed by drug exposure and viability assessments made the following day (15, 25–27). Cells were transfected with siRNA molecules or plasmids as described in prior manuscripts (20–24). Cells were transfected with a plasmid to express GFP-K-RAS V12 (0.1 μg) using lipofectamine 2000. Twenty-four hours after transfection, cells were used in assays examining their staining for GFP and RFP.

#### Detection of Cell Viability, Protein Expression, and Protein Phosphorylation by Immuno-Fluorescence Using a Hermes WiScan Machine [https://www.idea-bio.com/ (20–24)]

The text below discussing the Methods we use with the Hermes microscope is reproduced from text published in these review articles (28–30). “The Hermes machine combines high quality optics with a high-quality computer driven microscope stage, and with dedicated software, e.g., to analyze the immunofluorescent staining intensity of individual cells, i.e., *true* in-cell western blotting. A typical experiment: three independent cultures of a particular tumor cell type are sub-cultured into individual 96-well plates. Twenty-four h after plating, the cells are transfected with a control plasmid or a control siRNA, or with plasmids to express various proteins or validated siRNA molecules to knock down the expression of various proteins. After another 24 h, the cells are ready for drug exposure(s). At various time-points after the initiation of drug exposure, cells are fixed in place with permeabilization. Standard immunofluorescent blocking procedures are employed, followed by incubation of different wells with a variety of validated primary antibodies. The next morning, after washing, fluorescent-tagged secondary antibodies are added to each well; in general, we have found that using more than two tagged antibodies in each well-results in poorer data/image quality. After 3 h of incubation, the secondary antibody is removed, the cells washed again, and are hydrated with phosphate buffered saline prior to microscopic examination. Based on the experiment, cells are visualized at either 10X magnification for bulk assessments of immunofluorescent staining intensity or at 60X magnification for assessments of protein or protein-protein co-localization (Supplemental Figure 1).”

“For studies at 10X magnification, the operator selects which fluorescent antibody will be assessed first, i.e., in the red or green channel, and then focuses the microscope in a vehicle control transfection control well. The operator then outlines for the computer controlling the microscope “what is a cell.” In other words, the operator manually inputs the criteria for each specific tumor cell line segregating away detection of what is obvious debris or a staining artifact. The operator then sets how many cells per well are to be assessed for their immunofluorescent staining intensity; we initially selected 40 cells per well but have now moved to assessing 100. The computer/microscope then determines the background fluorescence in the well and in parallel randomly determines the mean fluorescent intensity of those 100 cells; the operator is provided with this mean intensity value. Of note for scientific rigor is that the operator does not personally manipulate the microscope to examine specific cells; the entire fluorescent accrual method is independent of the operator. Once the entire plate has been scanned for one of the secondary antibodies, the second secondary antibody with a different fluorescence range can similarly be used to define the mean intensity value in each well. Once data from the first set of plated cells has been obtained, the second and third sets of plated cells can be processed through the machine. Thus, we obtain three independent sets of fluorescence data from the three individual cultures, with 300 cells under each condition being assessed. Typically, the total expression of a particular protein will be assessed alongside additional staining to define the levels of different phosphorylation sites within the protein, e.g., total ULK1, P-ULK1 S317, P-ULK1 S757. Within these analyses it is also essential to include wells to define invariant protein loading controls, such as the expression level of ERK2 or AKT. For phospho-proteins, data can be presented in two ways, either bar graphs where the total protein expression/loading is presented alongside changes in phospho-protein levels, or with just bars for the phospho-protein fluorescence data corrected for the amount of protein expression, i.e., the stoichiometry of protein phosphorylation. For proteins whose total expression changes after drug exposure, the use of invariant total ERK2 or total AKT expression is used instead as a loading control. Usually, alongside our numeric bar graph data, we also present images of stained cells, taken at 60X magnification, which visually reveal the extent of protein over-expression or of protein knock down. The Hermes microscope has also proved very useful at examining protein-protein interactions at 60X magnification. Three to four images of cells stained in the red and green fluorescence channels are taken for each treatment/transfection/condition. Images are ~4 MB sized files. Images are merged using Adobe Photoshop. The image intensity and contrast is then *post-hoc* altered in an identical fashion inclusive for each group of images/treatments/conditions, so that the image with the weakest intensity is still visible to the naked eye for publication purposes but also that the image with the highest intensity is still within the dynamic range, i.e., not over-saturated.”

“At present, many laboratories still utilize traditional western blotting with secondary antibodies conjugated to luciferase, with enhanced chemiluminescence and X-ray film as a read-out; this approach has a limited dynamic range and for the 21st Century lacks sufficient rigor. Other laboratories with access to fluorescent imagers such as the Odyssey system use SDS PAGE with fluorescent tagged secondary antibodies, and these systems have a 5- to 6-log dynamic range. The Odyssey system can, at a gross level, also perform in-cell immunoblotting including co-staining in the red and green fluorescence channels. All of the above procedures require a considerable amount of operator input, including the isolation, lysis, clarification, and loading of proteins onto an SDS PAGE gel, followed by transfer to immobilon. This creates inherent errors in defining small drug-induced alterations to expression and phosphorylation; these are all processing stages where the rigor of the experiment can be compromised. Our use of the Hermes system abolishes all of the intermediate steps as cells are fixed *in situ*. Furthermore, unlike traditional SDS PAGE, the proteins retain their native conformations which for a number of proteins, e.g., detecting changes in chaperone conformation cause by drug exposures, presented data that could not have been obtained using traditional SDS PAGE. Thus, the in-cell assessments of altered phosphorylation or expression using the Hermes microscope provide data with more rigor and a much lower standard deviation difference, permitting changes of 20–30% intensity to be assessed for statistical significance.”

“An additional benefit of using the Hermes system is that it promotes a greater level of rigorous non-manipulatable data. As mentioned earlier, the cells are fixed in place and stained, and then once the machine has been set to recognize the morphology of any specific tumor cell type, the role of the operator has ended. Data is obtained by the machine in a random fashion examining cell staining intensities wherever it detects cells; the operator cannot skew their data by plating more cells in one well-compared to another, or by picking certain cells to scan, leaving other cells out. We believe that our approach using the Hermes WiScan microscope, or with similar computer-controlled microscope products, should become the standard of approach for immunoblotting/immunofluorescence work.”

#### Assessment of ROS

“Cells were treated with the drugs and 15 min prior to the indicated time point the media was removed and cells incubated with diacetate dihydro-DCF-DA (5 μM) (20–24). Fluorescence measurements were obtained 15 min after DCFH-DA addition with a Vector 3 plate reader. Data are presented corrected for basal fluorescence of vehicle-treated cells at each time point and expressed as the arbitrary units provided by the plate reader/the increase in ROS levels.”

#### Animal Studies

Studies were performed under VCU IACUC protocol AD20008. Animals, *n* = 3 per group, were treated for 14 days with vehicle control (cremophore QD Days 1–14) or with [fingolimod, FTY 0.6 mg/kg + DMF, 75 mg/kg]. For both drugs, this represents an approximate 3-fold higher dosing than would occur in a human multiple sclerosis patient. Five-micron sections of normal tissues were obtained, and H&E staining performed to detect any changes in tissue morphology. No alteration in animal body mass was observed comparing vehicle control treated and [FTY + DMF] treated mice (not shown).

### Data Analysis

Comparison of the effects of various treatments (in triplicate three times) was using one-way ANOVA and a two tailed Student's *t*-test. Statistical examination of *in vivo* animal survival data utilized a two tailed Student's *t*-test and log rank statistical analyses between the different treatment groups. Differences with a *p* < 0.05 were considered statistically significant. Experiments are the means of multiple individual points from multiple experiments (± SEM).

## Results

**Figure 1:**
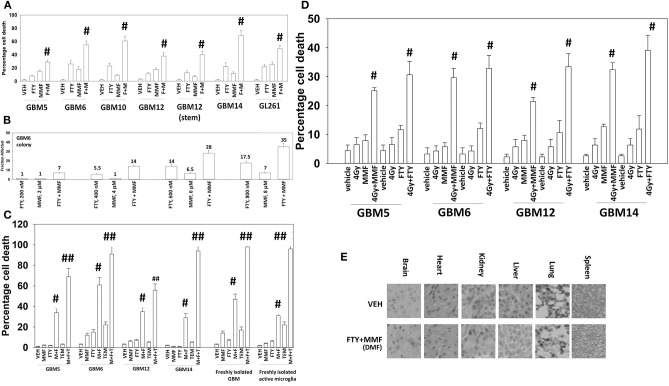
Gilenya and Tecfidera interact to kill GBM cells. **(A)** GBM cells were treated with vehicle control (VEH), fingolimod (FTY, 100 nM), MMF (5 μM), or the drugs in combination for 24 h. Cells were isolated, and viability determined by trypan blue exclusion assay (n = 3 ± SD) #*p* < 0.05 greater than fingolimod alone. **(B)** GBM6 cells were plated as single cells 250/500 cells per 60 mm dish. Twelve hours after plating, cells were treated with vehicle control, fingolimod (200–800 nM), MMF (2–8 μM), or the drugs in combination at a fixed dose ratio for 24 h. After 24 h, the media was removed, the cells washed with warm drug-free media, and then incubated in drug-free media for an additional 9 days. Cells/colonies were fixed in place and stained with crystal violet. Colonies of >50 cells were counted, and the fraction affected determined (*n* = 6 plates per-condition ± SD). **(C)** GBM cells and fresh activated microglia were treated with vehicle control [FTY, 100 nM + MMF, 5 μM], temozolomide (TMZ, 1 μM), or the drugs in combination for 24 h. Cells were isolated, and viability determined by trypan blue exclusion assay (*n* = 3 ± SD) #*p* < 0.05 greater than vehicle control; ##*p* < 0.05 greater than [FTY + MMF] value. **(D)** GBM cells were treated with vehicle control [FTY, 100 nM + MMF, 5 μM], ionizing radiation (4 Gy), or the drugs in combination for 24 h. Cells were isolated, and viability determined by trypan blue exclusion assay (n = 3 ± SD) # greater than radiation single agent value. **(E)** Mice were treated for 14 days QD and PO with vehicle control (cremophore) or with [fingolimod, FTY 0.6 mg/kg + DMF, 75 mg/kg]. Five-micron sections of normal tissues were obtained, and H&E staining performed to detect any changes in tissue morphology. No alteration in animal body mass was observed comparing vehicle control treated and [FTY + DMF] treated mice (not shown).

The Mayo clinic-derived and characterized PDX GBM cells are grown in bulk *in vivo* to maintain their tumorigenic biology and invasive characteristics, and the present studies used multiple isolates from different GBM5, GBM6, GBM12, and GBM14 tumors/mice *in vitro*. Fingolimod and MMF at physiologic concentrations interacted in an additive to greater than additive fashion to kill a genetically diverse group of primary and established GBM isolates and GBM stem cells (Figure 1A). Similar killing data were obtained using colony formation assays (Figure 1B). The combination of [MMF + fingolimod] enhanced the lethality of the standard of care drug temozolomide against GBM cells, and also against a fresh primary GBM isolate with its associated activated microglia (Figure 1C). Prior studies have demonstrated that the [MMF + fingolimod] combination could kill other primary GBM isolates, TNBC breast cancer cell lines, NSCLC isolates, ovarian isolates and sarcoma isolates (15). Both MMF and fingolimod radiosensitized our GBM isolates (Figure 1D). We next determined whether animals exposed to fingolimod and MMF in combination would exhibit any normal tissue toxicities. Animals were treated daily for 14 days with drug doses ~3 times higher than those of either drug in multiple sclerosis patients. Supra-physiologic doses of the drugs *in vivo* did not cause damage to “normal tissues” in the mouse (Figure 1E). This finding argues that the [MMF + fingolimod] drug combination will likely be “safe” in a human cancer patient.

We selected the PDX models GBM6 and GBM14 for further in-depth analyses. The GBM6 isolate expresses the truncated constitutively active ERBB1 vIII and the GBM14 isolate lacks PTEN expression. In many prior experimental therapeutics studies, we have performed agnostic screening analyses following exposure of cancer cells to drugs using the Hermes wide-field microscope. Using fluorescence intensity imaging of individual cells, we define the changes in the expression and phosphorylation of multiple signal transduction proteins (20, 21, 24, 31). Signaling by ERK1/2, AKT, mTOR, p70 S6K, STAT3, and STAT5 was assessed, as was signaling by ATM, the AMPK and eIF2 alpha. Studies also closely examined the regulation of autophagy, measuring the levels of Beclin1, ATG5 and the phosphorylation of ULK1 and ATG13. The levels of multiple cytoprotective proteins were assessed including MCL-1, BCL-XL, and c-FLIP-s.

**Figure 2:**
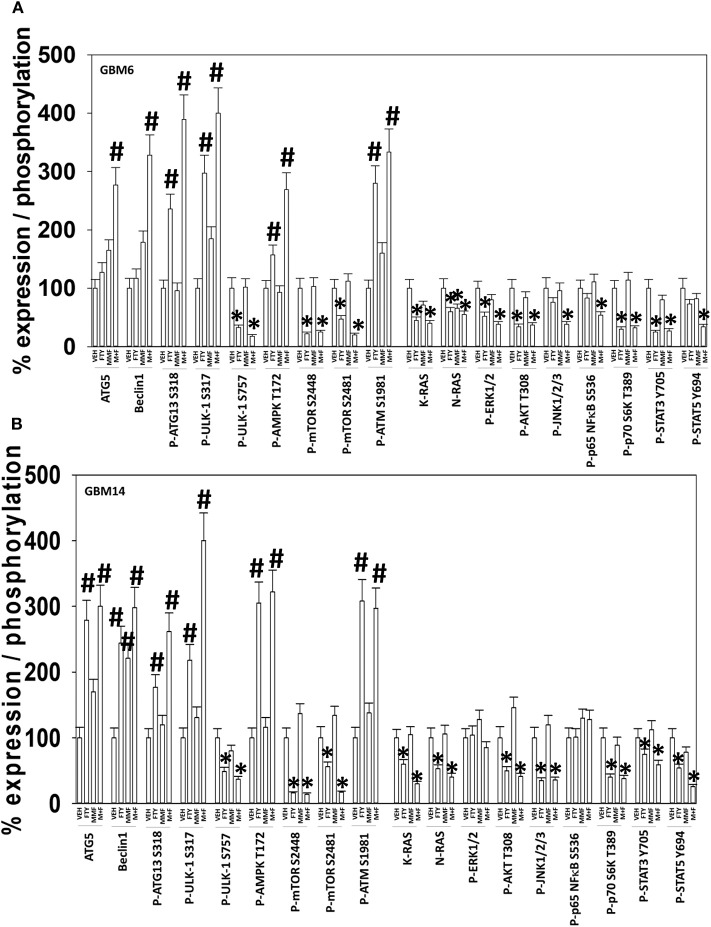
[FTY + MMF] exposure enhances ER stress signaling, causes DNA damage, and inactivates multiple protective signaling pathways. **(A)** GBM6 and **(B)** GBM14 cells were treated with vehicle control (VEH), fingolimod (FTY, 100 nM), MMF (5 μM), or the drugs in combination for 6 h. Cells were fixed in place and immuno-staining performed to detect the total expression and phosphorylation of the noted proteins. Analyses for densitometric scanning of fluorescence intensity were performed at 10X magnification on >120 cells per condition, in a random fashion in a Hermes WiScan machine (*n* = 3 ± SD) *p < 0.05 less than exposure in vehicle control; #*p* < 0.05 greater than exposure in vehicle control.

In GBM6, but not GBM14, the drugs combined to further activate ATM and AMPK; in several prior studies using different drug combinations we have delineated an ATM-AMPK-ULK1-ATG13 pathway that promotes autophagosome formation (Figures 2A,B) (20, 21, 24, 31). In GBM14 cells, fingolimod as a single agent strongly activated ATM-AMPK signaling. In both cell lines FTY720 and MMF interacted to promote ULK-1 S317 phosphorylation; S317 is a site targeted by the AMPK and elevated S317 phosphorylation equates to ULK-1 kinase activation. This was associated with enhanced ATG13 S318 phosphorylation; elevated S318 phosphorylation is a key trigger event to initiate autophagosome formation. The [MMF + fingolimod] combination rapidly inactivated mTORC1, mTORC2, AKT, ERK1/2, p70 S6K, STAT3/5, and NFκB. Inactivation of mTORC1 and mTORC2 results in ULK-1 S757 dephosphorylation that also promotes ULK-1 kinase activity. In both GBM6 and GBM14 cells, the drugs interacted to reduce STAT5 Y694 phosphorylation and in one of the isolates STAT3 Y705 phosphorylation. In one isolate, the drugs also interacted to reduce NFκB S536 phosphorylation. As judged by elevated PKR-like endoplasmic reticulum kinase (PERK) phosphorylation and elevated eIF2α S51 phosphorylation, FTY720 caused a strong endoplasmic reticulum stress response. In both isolates, the drug combination also reduced the expression of the caspase 8/10 inhibitor c-FLIP-s, that potentially could facilitate death receptor signaling.

**Figure 3:**
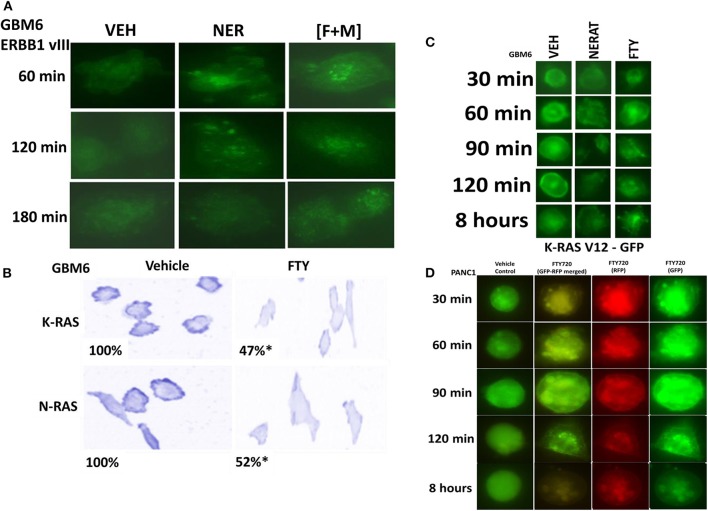
[FTY + MMF] exposure causes intracellular clustering of ERBB1 vIII and the degradation of RAS proteins. **(A)** GBM6 cells that express ERBB1 vIII were treated with vehicle control (VEH), neratinib (100 nM), or [fingolimod (FTY, 100 nM) + MMF (5 μM)] in combination. Cells were fixed in place at the indicated time points and the localization of ERBB1 vIII determined using an antibody raised against the COOH terminal portion of ERBB1. **(B)** GBM6 cells were treated with vehicle control (VEH) or with fingolimod (FTY, 100 nM) for 6 h. Cells were fixed in place and immuno-staining performed to detect the expression and localization of K-RAS and N-RAS. Analyses for densitometric scanning of fluorescence intensity were performed at 10X magnification on >120 cells per condition (*n* = 3 ± SD) *p < 0.05 less than exposure in vehicle control. **(C)** GBM6 cells were transfected with a plasmid to express K-RAS V12–GFP. Twenty-four hours later, cells were treated with vehicle control (VEH), fingolimod (FTY, 100 nM) or with the irreversible ERBB1/2/4 inhibitor neratinib (100 nM) for 0.5–8 h. Cells were imaged at 60X magnification and representative images from each condition at each time point are presented. **(D)** PANC1 pancreatic cancer cells that express an endogenous mutant K-RAS were transfected with plasmids to express K-RAS V12–GFP and K-RAS V12–RFP. Twenty-four h after transfection cells were treated with vehicle control or with fingolimod (FTY720, 100 nM) for the indicated time points. Cells were imaged at 60X magnification in the Hermes WiScan microscope and images were merged in Photoshop CS5.

We have recently demonstrated that the irreversible ERBB1/2/4 inhibitor neratinib could down-regulate the expression of RAS proteins, and based on this data and the fact that phosphorylated fingolimod causes sphingosine-1-phosphate receptor 1 internalization and degradation, we wished to determine whether this drug could act upon RAS proteins in GBM cells, in a manner similar to neratinib (20, 21, 24, 31). In GBM6 cells, that express an NH2-terminal truncated active ERBB1 vIII [fingolimod + MMF], caused intracellular clustering of the receptor; unlike fingolimod, our positive control neratinib increased clustering followed later by reduced expression of the receptor (Figure 3A). In GBM6 cells, fingolimod as a single agent could reduce the total expression of both wild type K- and N-RAS proteins (Figure 3B). To confirm this finding via a different approach, we transfected GBM6 cells with a plasmid to express K-RAS V12–GFP. In a manner similar to the data for ERBB1 vIII in Panel A, fingolimod caused intracellular vesicularization of K-RAS V12–GFP, that was maintained for 8 h (Figure 3C). In contrast, the positive control neratinib initially caused RAS vesicularization but at later times reduced the levels of GFP+ fluorescence. Finally, we wished to determine whether the effect of fingolimod on RAS vesicularization was specific only in GBM cells. PANC1 pancreatic cancer cells express a mutant K-RAS protein. PANC1 cells were transfected with plasmids to express K-RAS V12–GFP and K-RAS V12–RFP. In these cells, fingolimod appeared to cause greater levels of RAS vesicularization than were observed in the GBM cells (Figure 3D). In contrast to data from GBM cells, after 8 h of drug exposure, fingolimod reduced GFP+ and RFP+ fluorescence levels.

**Figure 4:**
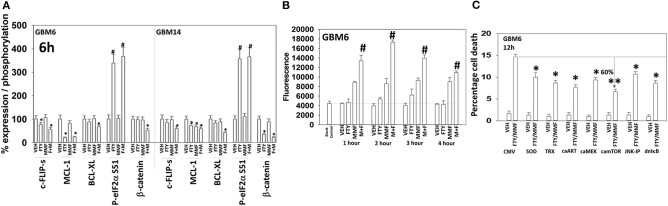
[FTY + MMF] exposure reduces the expression of c-FLIP-s, MCL-1, BCL-XL, and β-catenin and elevates reactive oxygen species levels. **(A)** GBM6 and GBM14 cells were treated with vehicle control (VEH), fingolimod (FTY, 100 nM), MMF (5 μM), or the drugs in combination for 6 h. Cells were fixed in place and immuno-staining performed to detect the total expression and phosphorylation of the noted proteins. Analyses for densitometric scanning of fluorescence intensity were performed at 10X magnification on >120 cells per condition (*n* = 3 ± SD). **(B)** GBM6 cells were treated with vehicle control, fingolimod (FTY, 100 nM), MMF (5 μM), or the drugs in combination. Fifteen min prior to the indicated time point the media was removed and cells incubated with diacetate dihydro-DCF-DA (5 μM). Fluorescence measurements were obtained 15 min after DCFH-DA addition with a Vector 3 plate reader. Data are presented corrected for basal fluorescence of vehicle-treated cells at each time point and expressed as the arbitrary units provided by the plate reader/the increase in ROS levels (*n* = 3 ± SD). **(C)** GBM6 cells were transfected with an empty vector control plasmid (CMV) or with plasmids to express SOD2, TRX, activated AKT, activated MEK1, activated mTOR, or IκB S32A S36A. The JNK-IP (10 μM) was added to a CMV transfected cell 30 min before any drug exposure. Twenty-four h after transfection, cells were treated with vehicle control or [fingolimod (FTY, 100 nM) + MMF (5 μM)] in combination for 12 h. Cells were isolated, and viability determined by trypan blue exclusion assay (*n* = 3 ± SD). ^#^*p* < 0.05 greater than corresponding VEH value; *p < 0.05 less than corresponding VEH value; **p < 0.01 less than corresponding VEH value.

Additional descriptive studies were performed to further delineate the responses of GBM cells to [MMF + fingolimod]. After drug exposure, the expression of proteins generally considered to be protective against toxic stresses including MCL-1, BCL-XL, c-FLIP-s declined whereas the expression of proteins that facilitated autophagosome formation, ATG5 and Beclin1, increased (Figures 2A,B, 4A). The expression of β-catenin declined. High non-physiologic concentrations of DMF (>5 μM) have been proposed to increase the expression of NRF2 and HO-1 that are proteins which in a broad sense will act to suppress reactive oxygen species (ROS) production. ROS production is essential for the activation and activities of many different types of immune cell. Reactive oxygen also activates cytosolic ATM (32). Lower concentrations of MMF rapidly enhanced the production of ROS in GBM cells that remained constant for almost 4 h (Figure 4B). As a single agent fingolimod, also at a clinically relevant concentration (100 nM), modestly and transiently increased ROS production. When the drugs were combined, fingolimod significantly enhanced the ability of MMF to generate ROS over the 4-h time course. Over-expression of thioredoxin (TRX) or superoxide dismutase 2 (SOD2) quenched ROS production and significantly reduced [MMF + fingolimod] lethality, as did expression of activated MEK1, dominant negative IκB S32A S36A, and treatment with the JNK inhibitory peptide (Figure 4C, not shown). In cells treated with [MMF + fingolimod], despite exhibiting elevated ROS levels, no compensatory survival alterations in the expression of NRF2 or HO-1 were observed (not shown).

**Figure 5:**
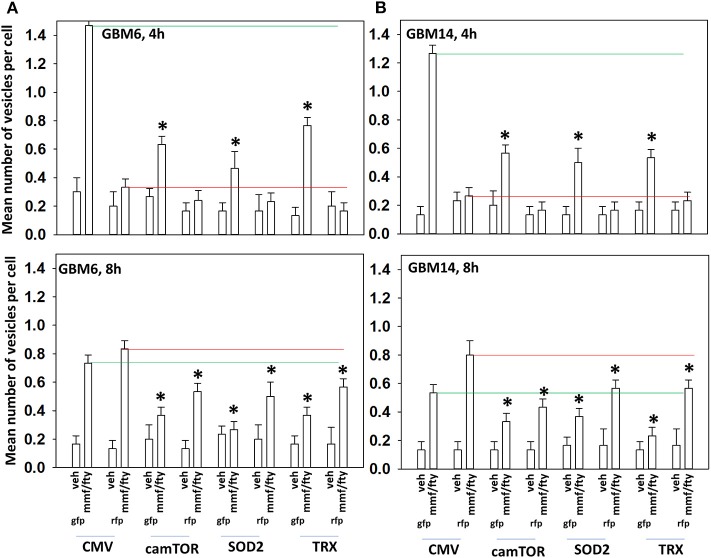
ROS production after [FTY + MMF] exposure is essential for autophagosome and autolysosome formation. **(A,B)** GBM6 and GBM14 cells were transfected with a plasmid to express LC3-GFP-RFP in parallel with an empty vector plasmid (CMV), a plasmid to express activated mTOR, or with plasmids to express superoxide dismutase 2 (SOD2) or thioredoxin (TRX). Twenty-four h afterwards, cells were treated with vehicle control (VEH) or with [fingolimod (FTY, 100 nM), MMF (5 μM)] in combination for 4 or 8 h. Cells were imaged at 60X magnification; at least 40 cells per condition in independent triplicate were examined and the mean number of vesicles per cell presented (*n* = 3 ± SD). *p < 0.05 less that corresponding value in CMV transfected cells.

In Figure 2 we observed that [MMF + fingolimod] inactivated mTOR and increased the phosphorylation of ATG13, all strongly suggesting that the drug combination was promoting autophagosome formation. GBM cells were transfected to express the fusion protein LC3-GFP-RFP which permits the detection over a time course of autophagosomes (GFP+ RFP+) and autolysosomes (RFP+); i.e., autophagic flux. Treatment of cells with [MMF + Fingolimod] increased the levels of autophagosomes followed temporally later by the formation of autolysosomes, arguing that the drug combination was stimulating autophagic flux (Figures 5A,B). Over-expression of TRX or SOD2 to quench reactive oxygen species or a mutant active form of mTOR to inactivate the kinase upstream of ATG13, ULK1, significantly reduced the drug-stimulated elevations in the levels of autophagosomes and autolysosomes.

**Figure 6:**
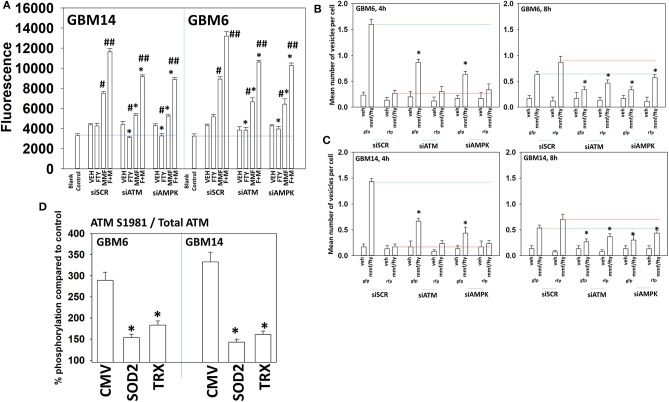
Activation of ATM and the induction of autophagy by [FTY + MMF] requires ATM-AMPK signaling. **(A)** GBM6 and GBM14 cells were transfected with a scrambled control siRNA (siSCR) or with siRNA molecules to knock down ATM or AMPKα. Twenty-four hours later, cells were treated with vehicle control or with [fingolimod (FTY, 100 nM) and MMF (5 μM)] in combination. Fifteen minutes prior to the indicated time point the media was removed and cells incubated with diacetate dihydro-DCF-DA (5 μM). Fluorescence measurements were obtained 15 min after DCFH-DA addition with a Vector 3 plate reader. Data are presented corrected for basal fluorescence of vehicle-treated cells at each time point and expressed as the arbitrary units provided by the plate reader/the increase in ROS levels (*n* = 3 ± SD) #*p* < 0.05 greater than corresponding vehicle control value; ##*p* < 0.05 greater than corresponding MMF value; *p < 0.05 less than corresponding value in siSCR cells. **(B,C)** GBM6 and GBM14 cells were transfected with a plasmid to express LC3-GFP-RFP in parallel with scrambled siRNA control (siSCR) or with siRNA molecules to knock down the expression of ATM or AMPKα. Twenty-four h later, cells were treated with vehicle control (VEH) or with [fingolimod (FTY, 100 nM), MMF (5 μM)] in combination for 4 or 8 h. Cells were imaged at 60X magnification; at least 40 cells per condition in independent triplicate were examined and the mean number of vesicles per cell presented (*n* = 3 ± SD). *p < 0.05 less that corresponding value in siSCR transfected cells. **(D)** GBM6 and GBM14 cells were transfected with an empty vector plasmid (CMV) or with plasmids to express superoxide dismutase 2 (SOD2) or thioredoxin (TRX). Twenty-four hours afterwards, cells were treated with vehicle control (VEH) or with [fingolimod (FTY, 100 nM), MMF (5 μM)] in combination for 6 h. Cells were fixed in place and immuno-staining performed to detect the total expression and phosphorylation of ATM S1981. Analyses for densitometric scanning of fluorescence intensity were performed at 10X magnification on >120 cells per condition (*n* = 3 ± SD) *p < 0.05 less than exposure in vehicle control; #*p* < 0.05 greater than exposure in vehicle control.

ATM in the cytosol can be activated by reactive oxygen species whereas ATM associated with DNA in the nucleus can be activated by DNA damage (32). Figure 2 demonstrated that fingolimod activated ATM but data in Figure 4 demonstrated that fingolimod weakly elevated reactive oxygen species levels. This suggests fingolimod may be causing a DNA damage-induced activation of ATM. And, as an HDAC inhibitor, fingolimod has the potential to cause DNA damage. Knock down of ATM or AMPKα modestly, though significantly, reduced amount of ROS generated by MMF and by [MMF + fingolimod] 2 h after exposure, each by ~20% (Figure 6A). Knock down of ATM or AMPKα significantly reduced the ability of the drug combination to stimulate autophagosome formation after 4 h by 45–90% (Figures 6B,C). Although evidence from Figures 2, 4 would argue that reactive oxygen species plays a secondary role in the regulation of ATM activity, over-expression of TRX or SOD2 significantly reduced the drug combination-stimulated phosphorylation of ATM S1981 ~50–60% (Figure 6D). Collectively, the data in Figures 2–6 demonstrate that the initial sharp increase in ROS generation caused by [MMF + fingolimod] exposure is essential for robust ATM activation and for autophagosome formation, and that ROS generation is upstream of ATM activation and autophagy. Future studies will be required to explore the role of nuclear DNA damage in the activation of ATM after drug exposure.

**Figure 7:**
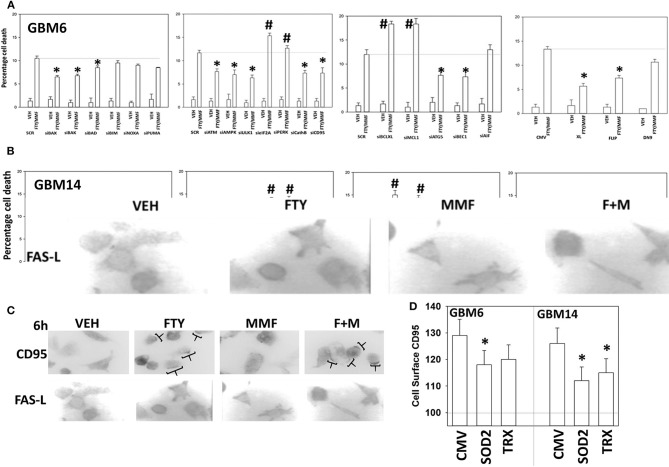
Knock down of CD95, ATM, AMPK, ULK1, ATG5, Beclin1, cathepsin B, BAX, and BAK suppressed [fingolimod + MMF] lethality whereas knock down of PERK, eIF2α, MCL-1, and BCL-XL promoted death. **(A,B)** GBM6 and GBM14 cells were transfected with a scrambled control siRNA (SCR) or with the siRNA molecules noted in the graph panels. In parallel, other portions of cells were transfected with an empty vector control plasmid (CMV) or with plasmids to express c-FLIP-s, BCL-XL, or dominant negative caspase 9. Twenty-four hours after transfection, cells were treated with vehicle control or [fingolimod (FTY, 100 nM) + MMF (5 μM)] in combination for 12 h. Cells were isolated, and viability determined by trypan blue exclusion assay (*n* = 3 ± SD). *p < 0.05 less killing than the corresponding value in both GBM6 and GBM14 cells; ^#^*p* < 0.05 greater killing than the corresponding value in both GBM6 and GBM14 cells. **(C)** GBM6 cells were treated with vehicle control, fingolimod (FTY, 100 nM), MMF (5 μM), or the drugs in combination for 6 h. Cells were fixed in place and immune-staining performed with validated antibodies to detect the total expression of CD95 and FAS-L. Cells were imaged at 60X magnification; areas in brackets indicate CD95 clustering on the plasma membrane. **(D)** GBM6 and GBM14 cells were transfected with an empty vector plasmid (CMV) or with plasmids to express superoxide dismutase 2 (SOD2) or thioredoxin (TRX). Twenty-four hours afterwards, cells were treated with vehicle control (VEH) or with [fingolimod (FTY, 100 nM), MMF (5 μM)] in combination for 6 h. Cells were fixed in place without permeabilization and immunostaining performed to detect CD95 cell surface localization. Cells were imaged at 60X magnification; at least 120 cells per condition in independent triplicate were examined and the mean number of vesicles per cell presented (*n* = 3 ± SD); *p < 0.05 less that corresponding value in CMV transfected cells.

Based on the data in Figures 1–6, using molecular tools, we next performed additional semi-descriptive studies designed ultimately to define the key protein regulators/pathways of viability after [MMF + fingolimod] exposure. Cell killing deliberately measured after only 12 h, i.e., the numeric values of percentage cell death are relatively low but are performed at this time point so as to define those key proteins/pathways who play a *primary* role in the killing processes. Proteins that were congruent in both PDX isolates for regulating tumor cell killing by the drug combination were: [BAX, BAK, BAD; MCL-1, BCL-XL] that could be considered as a mitochondrial apoptosis regulatory pathway; [ATM, AMPK, ULK-1, ATG5, Beclin1, Cathepsin B] that could considered as an autophagy/lysosomal pathway; [CD95, c-FLIP-s] as a death receptor pathway feeding into the apoptosis pathway; and enhanced [PERK, eIF2α] ER stress signaling that promotes cell survival (Figures 7A,B). With respect to death receptor signaling, fingolimod, but not MMF, caused plasma membrane clustering of CD95, indicative of death receptor activation (Figure 7C). Over-expression of the reactive oxygen species quenching enzymes TRX or SOD2 modestly, though significantly, reduced CD95 plasma membrane levels (Figure 7D). Tyrosine phosphorylation of CD95 is known to play a key role in its activation (33). Tyrosine phosphatases are potently inhibited by reactive oxygen species and thus inhibition of PTPases by ROS may represent a mechanism of CD95 activation.

**Figure 8:**
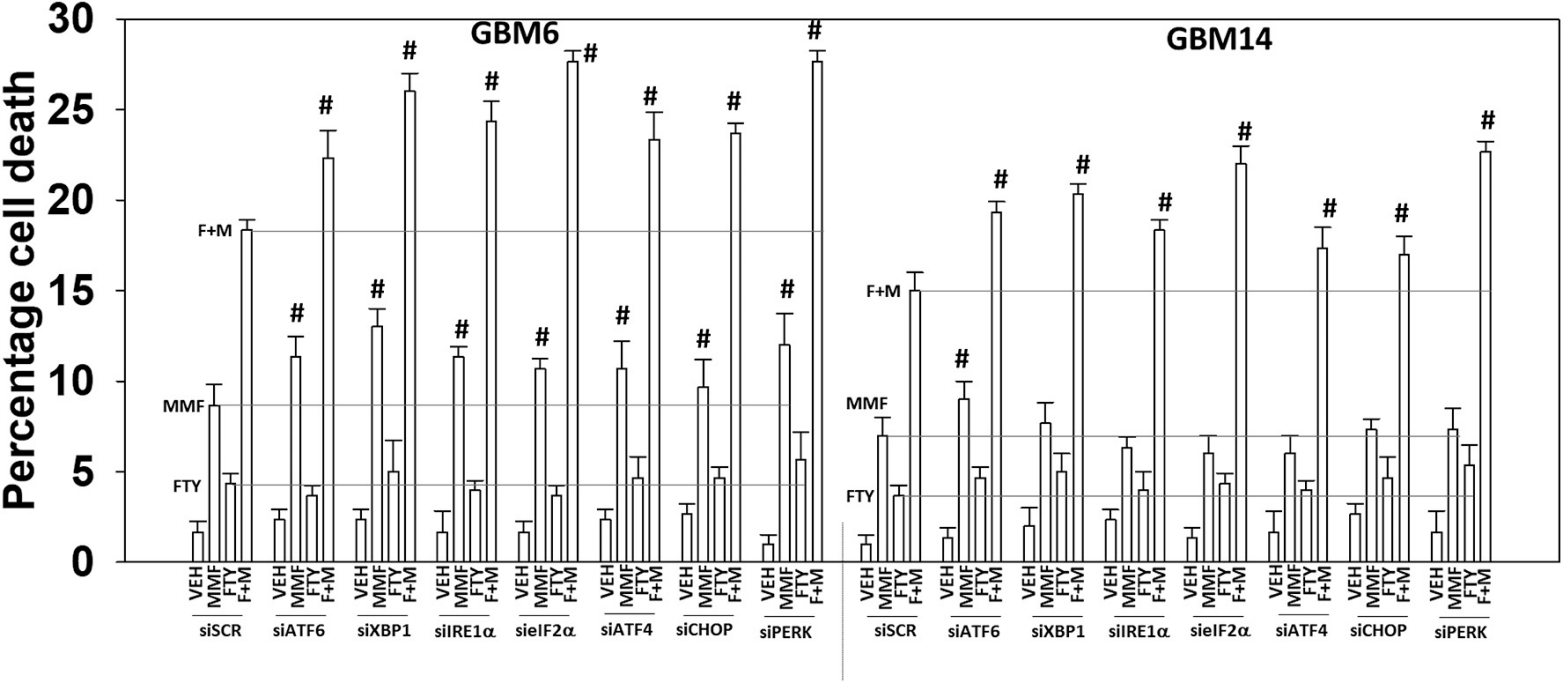
Endoplasmic reticulum stress signaling plays a protective role against [MMF + fingolimod] exposure. GBM6 and GBM14 cells were transfected with a scrambled siRNA control (siSCR) or with the indicated siRNA molecules to knock down the expression of proteins that regulate ER stress pathways. Twenty-four hours after transfection, cells were treated with vehicle control, MMF (5 μM), fingolimod (100 nM), or the drugs in combination for 12 h. Cells were isolated, and viability determined by trypan blue exclusion assay (*n* = 3 ± SD). #*p* < 0.05 greater than corresponding value in siSCR cells.

As presented in Figure 2, knock down of PERK or eIF2α enhanced [MMF + fingolimod] lethality, implying endoplasmic reticulum stress signaling was protective. Hence, we next determined whether other endoplasmic reticulum stress pathways regulated the survival response to [MMF + fingolimod] treatment. Knock down of the IRE1-XBP1 pathway or the ATF6 pathway, in a manner like knock down of the PERK-eIF2α pathway, enhanced killing GBM6 and GBM14 cells (Figure 8). In GBM6 cells, knock down of the ER stress pathways enhanced MMF lethality, whereas in GBM14 no enhancement occurred. In GBM6 cells, fingolimod lethality was not altered by knock down of the ER stress pathways whereas in the GBM14 cells, killing was modestly enhanced. Whether PTEN functionality (GBM14) or ERBB1 vIII expression (GBM6) specifically alters the role of ER stress signaling in survival will require studies beyond the scope of the present manuscript.

**Figure 9:**
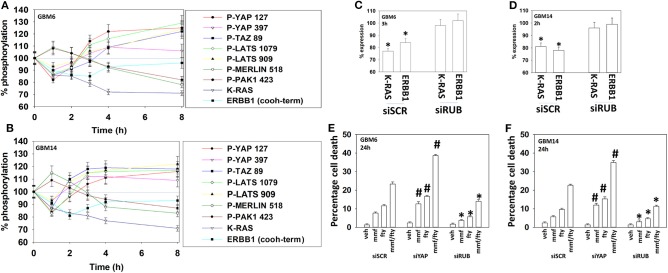
Fingolimod causes bi-phasic activation followed by inactivation of YAP and TAZ. **(A,B)** GBM6 and GBM14 cells were treated with vehicle control or with fingolimod (100 nM). At each time point over the 0–8 h time course cells were fixed in place and immunostaining performed to determine the fluorescence intensity levels of: total YAP, total TAZ; total Merlin; total PAK1; total LATS1; P-YAP S127; P-YAP S397; P-TAZ S89; P-LATS T1079; P-LATS S909; P-Merlin S518; P-PAK1 T423; total ERBB1; and total K-RAS from 10X magnification images (120 cells per condition, *n* = 3 ± SD). **(C,D)** GBM6 and GBM14 cells were transfected with a scrambled control siRNA (siSCR) or with an siRNA to knock down the expression of Rubicon. Twenty-four h after transfection cells were treated with vehicle control or with fingolimod (100 nM). At each time point over the 0–8 h time course cells were fixed in place and immunostaining performed to determine the fluorescence intensity levels of: total ERBB1; total K-RAS from 10X magnification images (120 cells per condition, *n* = 3 ± SD). *p < 0.05 less than vehicle control. **(E,F)** GBM6 and GBM14 cells were transfected with a scrambled control siRNA (siSCR) or with an siRNA to knock down the expression of Rubicon or the expression of YAP. Twenty-four h after transfection cells were treated with vehicle control, fingolimod (100 nM), MMF (5 μM), or the drugs in combination for 24 h. Cells were isolated, and viability determined by trypan blue exclusion assay (*n* = 3 ± SD). *p < 0.05 less than corresponding value in siSCR cells; #*p* < 0.05 greater than corresponding value in siSCR cells.

Glioblastoma is a highly invasive tumor type, and one signaling pathway that can interact with ERBB1-RAS signaling to promote tumor cell migration and invasion is the Hippo Pathway (34). The downstream effectors of this pathway, YAP and TAZ, are active when dephosphorylated, and in the nucleus where they act as co-transcription factors (35). Treatment of GBM6 and GBM14 cells caused a bi-phasic regulation of YAP and TAZ phosphorylation, as well as of their upstream kinases LATS1/2 and of docking proteins Merlin and PAK1 (Figures 9A,B). In both cell types, fingolimod within the first 2 h of exposure initially caused dephosphorylation of YAP, TAZ, and LATS1/2 and increased the phosphorylation of Merlin and PAK1. These events facilitate the co-transcription factor activities of YAP and TAZ, with Merlin acting to regulate complex formation and downstream signaling from small GTP binding proteins (36). At later times, 3–8 h after exposure, fingolimod caused the phosphorylation of YAP, TAZ, LATS1/2, and caused the dephosphorylation of Merlin and PAK1. Reduced Merlin phosphorylation enhances the ability of Merlin to act as a docking/chaperone protein bringing LATS1/2 into a closer association with YAP/TAZ, which enhances YAP/TAZ phosphorylation, thereby reducing YAP/TAZ activity (37).

One mechanism of plasma membrane internalization and subsequent protein digestion is called LC3-associated phagocytosis (LAP) (37). Key regulatory proteins in this process include Rubicon and Beclin1; our prior data demonstrated Beclin1 knock down protected cells from [MMF + fingolimod] lethality. Knock down of Rubicon prevented fingolimod from reducing the protein levels of K-RAS and ERBB1 (Figures 9C,D) (38). Knock down of Rubicon reduced the lethality of fingolimod, MMF and the drug combination (Figures 9E,F). In contrast, knock down of YAP enhanced killing by the drugs alone or in combination. These findings argue that reduced YAP phosphorylation caused by fingolimod leads to enhanced killing by the drug and that in cells incapable of LAP-dependent K-RAS/ERBB1 destruction, fingolimod lethality is significantly reduced.

## Discussion

The recognized actions of fingolimod and of DMF are to suppress immune system activity such that the host's auto-immune actions against demyelinated nerves, i.e., multiple sclerosis, are reduced. Although both drugs act to reduce the reactivity of the immune system, they do so through different mechanisms. Glioblastoma, for its overall malignancy and its invasion and privileged environment, relies heavily on its symbiotic relationship with activated/reactive microglia. As such, we hypothesized that a combination of the CNS-permeant drugs fingolimod and DMF/MMF could have therapeutic potential in this disease. Prior studies demonstrated that MMF reduced GBM cell invasiveness and enhanced the toxicity of temozolomide and ionizing radiation (15). MMF killed freshly isolated activated human microglia which correlated with reduced IL-6, TGFβ, and TNFα production. The MMF and fingolimod combination further reduced, below either agent individually, both GBM and activated microglia viability and their production of cytokines. In animals treated with DMF and fingolimod for 14 continuous days, no obvious damage to normal tissues was observed.

We demonstrated that the drug combination enhanced ATM/AMPK/ULK-1 signaling that corresponded with enhanced ATG13 S318 phosphorylation, as observed in prior therapeutics studies; in one isolate knock down of ATG5 and Beclin1 was protective against drug lethality whereas in the other it was not. Both isolates required the lysosomal protease cathepsin B for complete execution of the tumor cells. The drug combination reduced the protein levels of cytoprotective proteins such as ERBB1 vIII, K-RAS, N-RAS, c-FLIP-s, MCL-1, and BCL-XL. Knock down of CD95 or FADD, or over-expression of c-FLIP-s reduced drug combination killing arguing that the extrinsic pathway played a partial role in the killing process. Knock down of MCL-1 or BCL-XL enhanced tumor cell death whereas over-expression of BCL-XL was protective as was knock down of BAX, BAK and BAD. This data more definitively supports the drug combination causing mitochondrial dysfunction as a key component of the killing process. Knock down of apoptosis inducing factor significantly protected cells whereas expression of dominant negative caspase 9, i.e., “classic” apoptosis via caspase 3, did not. Alongside the role of cathepsin B, these findings demonstrate that the [MMF + fingolimod] combination kills through non-apoptotic processes.

At non-physiologic DMF/MMF concentrations, an order of magnitude higher than used herein, the drug has been shown to modulate the anti-oxidant response in cells, increasing the expression of NRF2 and HO-1. Because of this data linking DMF/MMF to the anti-oxidant response, we investigated whether MMF and fingolimod interacted to alter ROS levels and whether ROS generation play any role in tumor cell killing. MMF enhanced the production of ROS in GBM cells that was significantly enhanced in a greater than additive fashion by fingolimod. Over-expression of thioredoxin or superoxide dismutase 2 suppressed ROS production and drug combination lethality, yet the drug combination altered neither the levels of TRX and SOD2 expression, nor the levels of NRF2 and HO-1. Over-expression of either TRX or SOD2 significantly reduced the drug-induced phosphorylation of ATM, autophagosome formation and [MMF + fingolimod] lethality whereas the production of ROS was only marginally reduced in cells lacking ATM, CD95, or Beclin1. Thus, the greater than additive induction of ROS by the combination of MMF and fingolimod represents a key primary step in the initiation of the killing process. Further work will be required to define the source(s) of ROS production, e.g., mitochondria.

In many previous manuscripts we have demonstrated that a diverse set of compounds, and chemotherapeutic drug combinations, activate the death receptor CD95. For example, in primary hepatocytes, bile acid-induced CD95 activation can under certain circumstances enhance growth or, alternatively, cell death (39). In tumor cells, CD95 activation appears to only promote cell death. HDAC inhibitors, and fingolimod is an HDAC inhibitor, can increase the expression of CD95 and FAS-Ligand, and whilst [MMF + fingolimod] did not enhance CD95 and FAS-L levels, the drug combination did cause “capping” of CD95 on the cell surface. Genetically manipulated over-expression of TRX or SOD2 only partially reduced the plasma membrane clustering of CD95, arguing that other mechanisms play a more essential role in the process. Additional research will be required to fully define the molecular mechanisms of CD95 activation.

The molecular mechanisms by which [MMF + fingolimod] reduce the expression levels of K-RAS are poorly understood. Our data argued that the drugs reduced K-RAS and ERBB1 levels via Rubicon-dependent LAP followed by autophagic digestion. Clathrin-coated pits and caveolae are also two major endocytic structures which could play roles in RAS/ERBB1 destruction (40). Cholesterol, whose levels are regulated by AMPK signaling via inhibition of acetyl CoA carboxylase, an effect that also will lower the levels of farnesyl- and geranylgeranyl prenylation substrates, could impact K-RAS prenylation of K-RAS.

Fingolimod exposure initially “activated” YAP and TAZ by causing their dephosphorylation, followed later by enhancing their phosphorylation above baseline, i.e., reducing YAP/TAZ co-transcription factor activity. After fingolimod enters a cell it is phosphorylated where-after it acts in an autocrine fashion binding to S1P receptors. Initially, fingolimod causes receptor activation which is rapidly followed by receptor internalization and receptor degradation. S1P has been shown to reduce YAP phosphorylation and promote invasion, which is congruent with our findings (41, 42). However, when fingolimod has caused destruction of the S1P receptors, we observe increased YAP phosphorylation above basal levels. This suggests there may be a dynamic balance of S1P-dependent regulation of YAP in GBM cells which controls migration and invasion.

A considerable number of studies have used “modern” drug modulators of signal transduction processes in the hope of discovering a new approach to prolong survival of GBM patients. However, the median survival for GBM patients has only marginally improved over the past two decades when these drugs have been available. Checkpoint inhibitory and cellular immunotherapies have yet to exhibit any significant alteration in progression free or overall survival (43, 44). If we cannot enhance the immune system to attack GBM tumor cells, we reasoned, our initial conceptual approach became instead to attack the immune cell-rich soil in which the GBM tumor cells require to grow, thereby suppressing tumor cell growth and invasion, and with the hope that this will also enhance the lethality of standard of care therapeutics.

Our *in vitro* data has confirmed that the [MMF + fingolimod] drug combination acts to suppress the cytokine production and viability of freshly isolated activated human microglia. To our pleasant surprise, this drug combination also effectively killed multiple PDX isolates of human GBM cells, and both MMF and fingolimod enhance the lethality of temozolomide and of ionizing radiation. In addition to these observations was that this drug combination promoted the degradation of RAS proteins and oncogenic receptors such as ERBB1 vIII. Furthermore, the drugs inactivated YAP/TAZ signaling which further reduced viability. Future studies will be required to understand whether [MMF + fingolimod] can suppress GBM tumor growth *in vivo* in parallel with the combination altering cytokine production and modifying reactive microglia biology, without causing normal tissue toxicity.

## Data Availability Statement

All datasets generated for this study are included in the article/Supplementary Material.

## Ethics Statement

The animal study was reviewed and approved by Studies were performed per USDA regulations under VCU IACUC protocol AD20008.

## Author Contributions

PD wrote the manuscript. JH edited the manuscript and provided essential reagents. PD and AP conceptualized the experiments. LB and JR performed the experiments.

### Conflict of Interest

The authors declare that the research was conducted in the absence of any commercial or financial relationships that could be construed as a potential conflict of interest.

## Dedication

This manuscript is dedicated to the corresponding author's wife who for the past 6 years, daily, has had to take Gilenya.

## Supplementary Material

Supplemental Figure 1 Control data showing protein knock-down or protein over-expression in GBM cells. GBM6 cells were transfected to knock down or to over-express proteins (*n* = 3 ± SD).

## Abbreviations

ERK: extracellular regulated kinase
PI3K: phosphatidyl inositol 3 kinase
ca: constitutively active
dn: dominant negative
ER: endoplasmic reticulum
AIF: apoptosis inducing factor
AMPK: AMP-dependent protein kinase
mTOR: mammalian target of rapamycin
JAK: Janus Kinase
STAT: Signal Transducers and Activators of Transcription
MAPK: mitogen activated protein kinase
PTEN: phosphatase and tensin homolog on chromosome ten
ROS: reactive oxygen species
CMV: empty vector plasmid or virus
si: small interfering
SCR: scrambled
IP: immunoprecipitation
VEH: vehicle
DMF: dimethyl fumarate
MMF: monomethyl fumarate
FTY: FTY720, also known as fingolimod and Gilenya
HDAC: histone deacetylase
GBM: glioblastoma multiforme.
