# Corrigendum: Fingolimod Augments Monomethylfumarate Killing of GBM Cells

**DOI:** 10.3389/fonc.2020.00684

**Published:** 2020-04-30

**Authors:** Paul Dent, Laurence Booth, Jane L. Roberts, Andrew Poklepovic, John F. Hancock

**Affiliations:** ^1^Departments of Biochemistry and Molecular Biology, Virginia Commonwealth University, Richmond, VA, United States; ^2^Departments of Medicine, Virginia Commonwealth University, Richmond, VA, United States; ^3^Department of Integrative Biology and Pharmacology, University of Texas Health Science Center, Houston, TX, United States

**Keywords:** fingolimod, dimethyl fumarate, Gilenya, Tecfidera, RAS, glioblastoma, microglia

In the original article, there was a mistake in Figure 7C as published. By error, the image for FAS-L treated with MMF was included twice; once correctly and once as FAS-L treated with vehicle control. The corrected Figure 7C for FAS-L assessments appears below.

**Figure 7:**
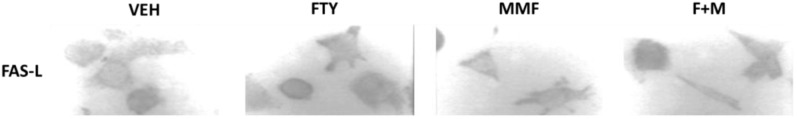


The authors apologize for this error and state that this does not change the scientific conclusions of the article in any way. The original article has been updated.

