# Supplementary figures and images for: Fingolimod Augments Monomethylfumarate Killing of GBM Cells
Source: Front Oncol. 2020 Jan 28;10:22. doi: 10.3389/fonc.2020.00022 (PMC6997152; doi:10.3389/fonc.2020.00022)

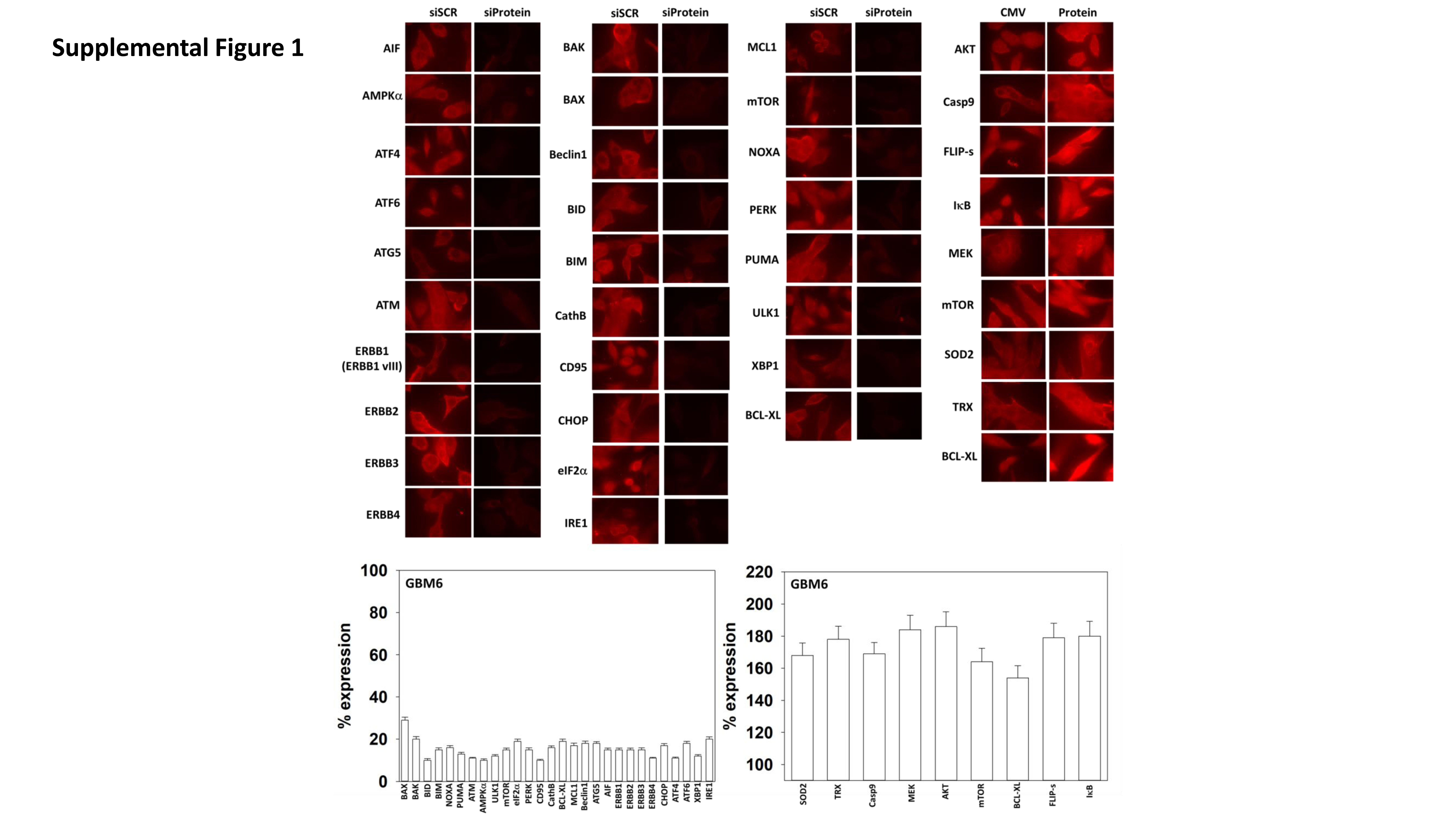

Supplement: Supplemental Figure 1 — Control data showing protein knock-down or protein over-expression in GBM cells. GBM6 cells were transfected to knock down or to over-express proteins (n = 3 ± SD). [file Image_1.TIF]
